# Faculty perspectives on factors influencing the passing of underperforming health profession students: challenging the ‘success-only’ culture

**DOI:** 10.3389/fmed.2026.1740755

**Published:** 2026-03-20

**Authors:** Hina Akhlaq, Sarwat Memon, Shahzad Memon, Muhammad Rizwan Memon, Muhammad Amber Fareed, Osama Khattak

**Affiliations:** 1 Prosthodontics Department, Institute of Dentistry, Liaquat University of Medical & Health Sciences, Jamshoro, Pakistan; 2 Obstetric and Gynaecology, Isra university hospital Hyderabad, Sindh, Pakistan; 3 Sindh Institute of Ophthalmic and Visual Sciences, Hyderabad, Pakistan; 4 Department of Prosthetic Dental Sciences, College of Dentistry, Jouf University, Sakakah, Saudi Arabia; 5 College of Dentistry, Ajman University of Science and Technology, Ajman, United Arab Emirates; 6 Center of Medical and Bio-Allied Health, Sciences Research, Ajman University, Ajman, United Arab Emirates; 7 Department of Restorative Dentistry, College of dentistry, Jouf University, Sakaka, Saudi Arabia

**Keywords:** assessment, dental education, faculty perspectives, failure to fail, medical education

## Abstract

**Introduction:**

In health professions, assessment plays an essential role in ensuring patient safety, professional competency and academic integrity. Nevertheless, in a variety of educational settings, the phenomenon of “failure to fail,” in which underperforming students are allowed to advance, has been documented. There is little data from Pakistan on how faculty members deal with this difficulty, especially in summative clinical and didactic evaluations. The purpose of this study was to investigate faculty perceptions of the variables affecting underperforming undergraduate students’ advancement in Pakistani health professions (medical, dental & allied).

**Methods:**

A cross-sectional mixed-methods design was employed using a semi-structured, anonymous Google form. The survey was distributed through professional faculty Whatsapp groups. Participation was voluntary and only faculty members actively involved in undergraduate assessments were eligible. Purposive sampling yielded 33 completed responses.

**Results:**

The majority of faculty (84.8%) stated that students who performed poorly were nevertheless able to pass. Institutional uncertainty (39.4%), social pressure (27.3%), and pity (12.1%) were among the major factors that contributed. Even though 78.8% had previous experience with failing students, the majority of their decision was based on academic underperformance (72.7%), considering the attitudes of faculty regarding failing students, one-third of participants acknowledged it to be a emotional turmoil, however the majority rejected the fact that failing a student was a reflection of their own teaching. There was little knowledge of institutional support systems (42.4%) and many respondents were either impartial or ignorant of the impact of organisational policies.

**Discussion:**

Despite faculty concerns, the majority of participants reported seeing instances where students with subpar or questionable performance advanced to later academic years. Commonly cited factors included uncertainty regarding institutional policies, limited remediation pathways, and emotional and ethical challenges associated with failing students. Qualitative analysis identified themes related to emotional burden, moral dilemmas, perceived institutional pressure, and patient safety concerns.

## Introduction

In medical education, it is essential to accurately and transparently assess student performance since inadequacies in this process can jeopardise academic integrity, patient safety, and student competency. Despite its significance, there are concerns that educators could potentially be allowing students pass in clinical and academic settings with subpar performance (1, 2). The medical profession’s ethos continues to be based on the ideas of ancient Greek philosophy, especially Hippocrates’ “Primum non nocere” (first, do no harm), which remains the foundational principle of medical ethics. The purpose of this aphorism is to balance beneficence and non maleficence in order to maximize benefit and minimize risk, not to emphasize one over the other (3, 4).

The phenomenon of “Failure to Fail” has been defined as faculty members’ reluctance to provide failing grades to students who are incompetent in didactic and clinical domains (5). Despite evidence of subpar performance, assessors may permit students to advance, which raises worries that unprepared people may enter the workforce and jeopardise patient safety (6). This problem has been documented in a number of professions that need certified practice, such as nursing (2, 7, 8), physiotherapy, medicine, and dentistry (9–12). Despite improvements in frameworks like competency-based medical education (CBME) and entrustable professional activities (EPAs) as well as assessment techniques like Objective Structured Clinical Examinations (OSCEs) and multiple-choice questions (MCQs), reluctance to fail students persists (13, 14). Research indicates that assessors often feel unprepared or hesitant when reporting inadequate performance. According to Yepes-Rios et al., the dynamics between supervisors and trainees, assessment culture and instruments, and institutional remediation protocols (15) are the primary barriers of failing underperforming trainees. Likewise, Chin et al. reported that even in situations where dismissal of trainees may be reasonable, supervisor discomfort and interpersonal ties have a substantial impact on assessment procedures (16).

Dentistry poses distinct challenges in contrast to other medical specialities (17–19). Even though dental care is typically provided in ambulatory settings, adverse outcomes remain possible, particularly for medically vulnerable patients. Unlike institutional medical settings, dental practices are more dispersed, isolated, and less structured, increasing the risk that negative outcomes go unrecognized and highlighting the need for stronger educational safeguards (20, 21).

Although there is wealth of literature on faculty experiences with “Failure to Fail” in nursing and other allied health fields (7, 8, 11, 12, 22–24), the phenomena has gotten relatively less attention, especially in dentistry education (25). Since underperformance by practitioners in dentistry can also jeopardise patient safety, public confidence, and professional reputations, this issue warrants focused investigation (12, 26).

This study aimed to investigate the phenomenon of “Failure to Fail” among undergraduate health profession students in Pakistan, focusing on summative clinical and didactic assessments. The research question guiding this study was: *What factors impact faculty decisions to pass underperforming undergraduate students in medical, dental, and allied health professions?* The study investigated faculty perceptions of the concept and their experiences assessing students with subpar clinical performance.

## Methodology

The study used a cross-sectional mixed-methods approach using a semi-structured survey distributed via anonymous Google forms to reduce any perceived risk to participants. Participation was voluntary, and informed consent was obtained electronically before respondents accessed the survey. No identifying information, including IP addresses, was gathered to ensure confidentiality. Ethical approval was granted by the Liaquat University of Medical and Health Sciences review board with letter number LUMHS/REC/−393.

For this study, a self-designed survey form was developed with item development conceptually guided by existing literature exploring ‘failure to fail’ behavior among examiners of undergraduate medical programs (27). The survey form comprised 20 items divided into three sections: 7 demographic questions, 11 Likert-scale items for quantitative assessment assessing faculty attitudes and institutional influences, and 2 open-ended questions for qualitative insights exploring participants’ understanding of the concept of “failure to fail and perceived challenges in assessing underperforming students.

Purposive sampling was employed to include participants using faculty WhatsApp groups that are often used for academic correspondence. Since the sampling technique demands the selection of participants with knowledge pertinent to the study’s objectives (28), only faculty members with past experience evaluating undergraduate students were included in the study. This method facilitated that the information gathered was contextually rich and directly related to evaluation procedures, even though it might restrict generalizability.

Quantitative data were analyzed using descriptive statistics in SPSS version 24, while qualitative responses were examined through thematic analysis to extract key themes from participants’ narratives.

## Results

### Participant characteristics

Table 1 summarizes the demographic characteristics of the participants. The majority of responders were female, between the ages of 31 and 40, and had been teaching for less than 10 years, with dentistry representing the largest discipline.

**Table 1:** Demographic characteristics of participants (*N* = 33).

Variable	Category	Frequency (*n*)	Percent (%)
Age	Below 30 years	1	3.0%
	31–40 years	23	69.7%
	41–50 years	8	24.2%
	51–60 years	1	3.0%
Gender	Male	13	39.4%
	Female	20	60.6%
Teaching experience	Less than 10 years	19	57.6%
	11–20 years	13	39.4%
	More than 20 years	1	3.0%
Academic discipline	Dental	28	84.4%
	Allied/basic	2	6.1%
	Medical	2	6.1%
	Other	1	3.0%
Academic position	Lecturer	14	42.4%
	Assistant Professor	10	30.3%
	Associate Professor	3	9.1%
	Medical/Dental officers	4	12.1%
	Professor	2	6.1%
Students evaluated per year	More than 150	12	36.4%
	51–100	11	30.3%
	101–150	7	21.2%
	Around 50	3	9.1%

### Faculty experience with failing students

The majority of respondents reported instances where students progressed in spite of performance issues. The responses concerning reasons for student progression are summarized in Table 2. While a lower percentage cited absenteeism or other reasons, a considerable proportion of faculty reported failing at least one student during their teaching careers, usually due to academic underperformance. The highest percentage (36.4%) of responders stated they had evaluated over 150 students / year. Most participants (75.8%) reported the proportion of students failed per year was less than 25%. Detailed description is presented in Table 2.

**Table 2:** Faculty experience with failing students.

Variable	Category	*n*	Percentage (%)
Students pass with questionable performance?	Yes	28	84.4%
	No	5	15.2%
Reason for passing an underperforming student	Institutional policies	13	39.4%
	Societal pressure	9	27.3%
	Sympathy	4	12.1%
	Ignorance/blind chance	3	9.1%
	Cultural factors	2	6.1%
	Personal reasons	2	6.1%
Personal experience of failing a student	Yes	26	78.8%
	No	7	21.2%
If yes, reason for failing a student	Underperformance in academics	24	72.7%
	Absenteeism	2	6.1%
	(Not applicable – No experience)	7	21.2%
Students evaluated per year	Around 50	3	9.1%
	51–100	11	30.3%
	101–150	7	21.2%
	More than 150	12	36.4%
Frequency of students failed/year	100%	2	6.1%
	25–50%	6	18.2%
	< 25%	25	75.8%

### Faculty attitudes toward failing students

There were differing opinions among faculty members on failed students. While many did not consider failing students as an indication of inadequate teaching or undermined professional integrity, some respondents (33%) acknowledged the emotional difficulty of giving failing grades. Faculty attitudes varied, as seen by differing opinions on the implications of workload (42.4%) and institutional strain (33.3%) Table 3.

**Table 3:** Faculty attitudes toward failing students.

Statement	Agree *n* (%)	Neutral *n* (%)	Disagree *n* (%)
Failing reflects poorly on my teaching	6 (18%)	13 (39%)	14 (42%)
Failing causes emotional distress	11 (33%)	19 (57%)	3 (9%)
Failing burdens institutional resources	11 (33.3%)	0 (0%)	22 (66.7%)
Failing is important for student long term development	4 (12.1%)	0 (0%)	29 (87.9%)
Failing compromises professional integrity	3 (9%)	0 (0%)	30 (90.9%)
Failing increases faculty workload	14 (42.4%)	0 (0%)	19 (57.6%)

When enquired if institutional policies and support systems influence decisions about failing students: *(Does institutional policies and support systems impact decisions about failing students?).* There was a great deal of ambiguity in the responses of participants. The impact of institutional policies on their failed decisions was not confirmed nor rejected by many faculty members. Participants’ awareness of available organisational support also varied; some claimed minimal awareness or no support at all (21.2%), while others acknowledged the existence of support structures (Table 4).

**Table 4:** Institutional influence on failure decisions.

Statement	Agree *n* (%)	No *n* (%)	Neutral/Unaware *n* (%)
Institutional policies influence failure decisions	3 (9.1%)	8 (24.2%)	22 (66.7%)
Awareness of organisational support	14 (42.2%)	7 (21.2%)	12 (6.4%)

**Figure 1:**
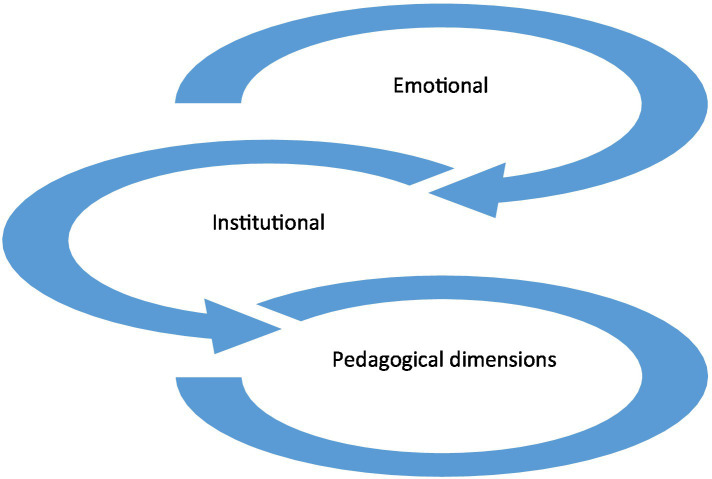
The intersecting viewpoints of term “failure to fail”.

Qualitative analysis first explored how faculty understood the phenomenon of “failure to fail”. Table 5 summarizes participants understanding and perceptions of the term below. Faculty members expressed various interpretations. Some associated it with an inability to assign a failing grade despite underperformance, while others highlighted institutional or cultural factors that constrained their ability to fail underperforming students. Participants also related the term with emotional strain, ethical dilemmas and fears regarding students’ futures. Overall, these perceptions were grouped into three overarching perspectives: emotional, institutional, and pedagogical. All together, the understanding of participants fall into three main overarching perspectives: emotional, institutional, and pedagogical (Figure 1).

**Table 5:** Faculty conceptualisations of the “failure to fail” phenomenon (qualitative themes and descriptions).

Theme	Description
Inability to fail underperforming students	Difficulty assigning failing grades despite concerns
Emotional and ethical conflict	Sympathy, fairness, and moral discomfort
Lack of academic standards or consequences	Perceived absence of clear failure thresholds
Institutional or systemic pressure	Political, administrative, or policy-related constraints
Conceptual ambiguity	Unclear or inconsistent understanding of the term
Philosophical framing	Viewing failure as developmental rather than punitive
No response	Did not comment on the concept

A second qualitative analysis examined the perspectives on the challenges in assessing student performance and the repercussions of failing students surfaced. Table 6 presents the nine essential themes that emerged through analysis of open-ended responses. Prominent themes included emotional and psychological strain on students including mental stress and depression, institutional or external pressures such as influence from families, authorities, institutions, and political considerations. Some other themes also emerged concerning patient and public safety, ethical challenges, career and academic ramifications for students especially the long-term effects on employment prospects and graduation delays, limitations within academic systems such as unfair academic systems, corruption, and a lack of autonomy, as well as reservations surrounding student behavior and professionalism.

**Table 6:** Perceived challenges in assessing underperforming students and consequences of failure (qualitative theme and description).

Theme	Description
Emotional and psychological burden	Depression, regret, lifetime regrets, mental stress, disturbing for student
Institutional or external pressure	Pressure from institute, family pressure, authority pressure, political influence
Concerns for patient/public safety	Patient safety, should not be harmful to society, empathy toward students
Career and academic consequences for students	Delays in graduation, impact on career prospects, destroy academic record, lagging behind peers
Unfair system/corruption/lack of autonomy	Corrupt academic system, incompetent policies, not enough support, political links
Misbehavior or professionalism issues	Misbehavior, students shout and blame teachers
Hope for future improvement	He improved later, better performance in next exam, positive impact in most cases
Ethical dilemmas and decision-making conflict	Balancing fairness, academic integrity vs. future aspirations, wrong message to society, ethical dilemma
No specific challenge reported	No challenges identified

### Integration of quantitative and qualitative findings

Quantitative findings demonstrated that many faculty members expressed emotional hardship and ambiguity surrounding institutional policies, while qualitative themes enlarged on these experiences by highlighting emotional strain, perceived administrative pressures, and concerns about patient safety. These results collectively demonstrate how decisions to pass underperforming students are influenced by both structural and personal variables. Together, these two aspects offer an in-depth insight into the phenomenon of failure-to-fail.

## Discussion

This study highlights that the phenomenon of “failure to fail” arises from a cumulative response of emotional, institutional, cultural, and ethical factors rather than being solely the product of individual hesitancy. The majority of faculty in our study acknowledged student progression despite their questionable performance, which raises concerns about academic standards and professional competence. These findings align with existing researches across multiple health professions, which also reflects on multidimensional influences on decision-making (8, 15). The cumulative conclusion of quantitative patterns and qualitative themes indicates that passing underperforming students is not a product of a single factor, but larger systemic and sociocultural dynamics.

A notable finding of the results was the emotional and psychological burden experienced by faculty when faced with failing decisions. Participants described assessment process as emotionally draining especially when they are required to strike a balance between ethical dilemmas and empathy, particularly in settings where decisions may be influenced by intimate professional connections. Faculty reported concerns regarding long-term distress to students’ mental resilience, learning development with time, and career prospects, along with reservation regarding patient safety. These emotionally charged presumptions align with prior research describing assessors’ difficulty of being unsupportive or damaging to students’ self-esteem (10, 15). Such findings strongly supports the facts that assessment decisions are deeply embedded in ethical and relational contexts.

Institutional factors further impact the decisions. Faculty responses shows substantial ambiguity uncertainty regarding assessment guidelines and institutional support mechanisms. Inconsistent policies and assessment structures as well as lack of confidence in administrations surfaced as the few reasons behind faculty’s resistance to assign failing grades. Additionally, participants also reported more hesitance toward lack of clarity in institutional processes than excess pressure to pass students. This finding is in line with researches demonstrating that dubious policies, fear of administrative repercussions, and poor leadership support impact assessors from failing underperforming students (8, 15). Although the current study ensured anonymity of participants, institutional influences may have been underreported due to the sensitivity of the topic, representing a potential limitation of the study.

Another significant factor surfaced regarding educational and regulatory context in results. In Pakistan, undergraduate dentistry education usually follows a hybrid curriculum model that blends conventional methods with specific competency-based components, like objective structured clinical examination (OSCEs) and structured clinical assessments. In the absence of a fully adopted competency-based medical education framework with nationally standardized benchmarks, assessors may rely significantly on institutional standards, clinical exposure, and professional subjective judgement. In the Pakistani context, where assessment frameworks and remedial procedures may differ significantly between institutions, the lack of uniform national guidelines may increase assessor uncertainty.

These obstacles may also be affected by variability in assessment tools and the absence of standardized rubrics across institutions, which could result in assessor uncertainty.

Cultural and societal expectations were prominent in faculty reflections with participants complaining about broader social norms such as familial pressures and community relationships. In such contexts, allowing progression may be seen as socially protective rather than professional standpoint (29). These sociocultural and institutional pressures influence a ‘success-only culture,’ where student progression is given precedence over remediation or justified failure, potentially compromising rigorous assessment standards. The current findings highlight the importance of understanding evaluation procedures in the context of sociocultural context rather than considering “failure to fail” as a personal or technical shortcoming.

Participants reported that they had the confidence to make challenging assessment decisions when performance inadequacies were deemed substantial, suggesting that the “failure to fail” issue is neither absolute nor inevitable. These choices were heavily influenced by considerations of patient safety, professional accountability, and ethical responsibility, which are consistent with qualitative research and worldwide literature in the fields of medical, nursing, and dentistry education (8, 10, 15). Faculty narratives further demonstrated how these self-assured choices were tempered by institutional support, emotional strain, and possible repercussions for students, highlighting the intricate interplay of societal, professional, and personal variables in poor decisions.

Taken together, these findings indicate the need for structural and cultural reform. Clear institutional policies, transparent assessment criteria, strong leadership support along with faculty development can somehow overcome the uncertainty and emotional burden amongst the faculty. Failing a student should be viewed as a shared professional responsibility among educators, institutions, and assessment systems rather than as an ethical lapse or a decision made by a single instructor. Educators are more likely to make just, consistent decisions that put patient safety and student learning foremost when they have clear policies and institutional support (7, 12). The term “success-only culture” describes an educational setting where passing students are tacitly given priority, frequently at the price of honest evaluation and remediation. In order to uphold academic integrity, promote the progression of competent and maintain the public confidence in health professions, this culture must be addressed.

These results are consistent with research conducted worldwide, but they also emphasize the significance of contextual awareness, especially in South Asian educational systems. To guarantee that only qualified graduates advance and uphold professional standards and public trust, it is essential to strengthen faculty training, improve peer support, and create cohesive institutional frameworks.

## Conclusion

This study emphasizes that institutional ambiguity, emotional strain, and social pressures—rather than just personal hesitancy—are the main causes of the “failure to fail” issue among health professions faculty in Pakistan. Only a small percentage of teachers reported clarity or support from institutional policies, with many being either oblivious, indifferent, or skeptical of their impact, despite the fact that the majority identified situations in which underperforming students were passed. These results highlight the critical need for clear assessment norms, structured remediation procedures, and robust leadership support to increase faculty confidence in conducting impartial assessments. Crucially, educators may experience less emotional conflict if failure is reframed as a professional obligation rather than a personal weakness. Institutions can foster the progression of competent graduates by filling in these systemic weaknesses, protecting patient safety, academic integrity, and public safety.

### Limitations

When evaluating the results, several limitations should be accounted. The data were gathered from a single institutional context and the sample size was modest (*n* = 33), which restricts the results’ applicability in different contexts. Self-reported responses may reflect respondent misunderstanding especially in Table 1 and should be interpreted with caution. Additionally, the institutional influences may have been underreported due to the sensitivity of the topic, representing a potential limitation of the study. The participant pool was skewed toward lecturers and early- to mid-career faculty because fewer senior academics took part. The opinions recorded may have been impacted by this distribution, especially when it came to making decisions about failed students. But it also represents those who are most actively engaged in the institution’s regular undergraduate assessment. Therefore, rather than being representative of all academic roles or institutions, the results should be considered as exploratory and illustrative of faculty experiences. The questionnaire’s lack of psychometric validation limits the generalisability of our results beyond the study population. Lastly, the lack of formalized assessment frameworks, which may also impact decision-making, is reflected in the study’s failure to gather data on self-assessment, rater reliability, rubrics, moderation, supervision duration, and assessment timing. Future studies employing bigger, multi-institutional samples with more equitable representation across academic ranks and years of experience would be beneficial to clarify whether perspectives differ by role or seniority.

## Data Availability

The raw data supporting the conclusions of this article will be made available by the authors, without undue reservation.
